# Modulating the Oxytocin System During the Perinatal Period: A New Strategy for Neuroprotection of the Immature Brain?

**DOI:** 10.3389/fneur.2018.00229

**Published:** 2018-04-13

**Authors:** Manuela Zinni, Marina Colella, Aline Rideau Batista Novais, Olivier Baud, Jérôme Mairesse

**Affiliations:** ^1^INSERM U1141 Protect, Paris-Diderot University, Paris, France; ^2^Neonatal Intensive Care Unit, Robert Debré Children’s Hospital, Paris, France; ^3^University of Geneva, Geneva, Switzerland; ^4^Division of Neonatology, Geneva Children’s Hospital, Geneva, Switzerland

**Keywords:** intra-uterine growth restriction, neuro-inflammation, white matter brain injury, oxytocin, microglia, glucocorticosteroid, GABA, maternal behavior

## Abstract

Oxytocin is a neurohypophysal hormone known for its activity during labor and its role in lactation. However, the function of oxytocin (OTX) goes far beyond the peripheral regulation of reproduction, and the central effects of OTX have been extensively investigated, since it has been recognized to influence the learning and memory processes. OTX has also prominent effects on social behavior, anxiety, and autism. Interaction between glucocorticoids, OTX, and maternal behavior may have long-term effects on the developmental program of the developing brain subjected to adverse events during pre and perinatal periods. OTX treatment in humans improves many aspects of social cognition and behavior. Its effects on the hypothalamic–pituitary–adrenal axis and inflammation appear to be of interest in neonates because these properties may confer benefits when the perinatal brain has been subjected to injury. Indeed, early life inflammation and abnormal adrenal response to stress have been associated with an abnormal white matter development. Recent investigations demonstrated that OTX is involved in the modulation of microglial reactivity in the developing brain. This review recapitulates state-of-the art data supporting the hypothesis that the OTX system could be considered as an innovative candidate for neuroprotection, especially in the immature brain.

## White Matter Injury (WMI) Following Fetal Growth Restriction

Intrauterine growth restriction (IUGR) is a complication observed in 10% of the pregnancies (1) and represents the major causes of neonatal mortality and morbidity (2). Placental insufficiency resulting in fetal hypoxia and maternal malnutrition are two identifiable and major causes of IUGR (3). Due to its constant increase in both industrialized and developing countries, where 2.8 million children out of 135 million born in 2010 were born preterm and growth restricted (4), IUGR represents an important public health problem. Indeed, growth-restricted infants showed a higher risk of perinatal morbidity and of neurodevelopmental alteration with long-term cognitive and neurobehavioral handicaps (5, 6). Interestingly, studies based on magnetic resonance imaging have clearly evidenced that the cognitive and psychiatric deficits observed (7–9) are correlated to alterations of brain white and gray matter (7, 10, 11), including altered neural circuitry (12, 13). The importance of IUGR in the context of public health economy is further highlighted by the presence of a positive correlation between the severity of IUGR and the risk to develop cerebral palsy (14), risk that is 10- to 30-fold higher in IUGR babies (15–18).

The mechanisms responsible for the induction of brain injury in preterm infants remain largely elusive and several putative inductor factors have been identified (oxidative stress, excitotoxicity, neuroinflammation) (19). In the hereinafter of this section, we will focus on the role of neuroinflammation and the possible cellular mechanisms responsible for inflammatory-induced brain damage.

Clinical studies showed that abnormal inflammatory responses in the fetus and/or in the neonate can contribute to white matter damage (20, 21). These clinical observations are well supported by studies conducted in rodents in which IUGR is not only associated with an abnormal neuroinflammatory response and myelinization defects (22–24), but is also a risk factor for the development of inflammatory-induced brain damage (25).

**Figure 1:**
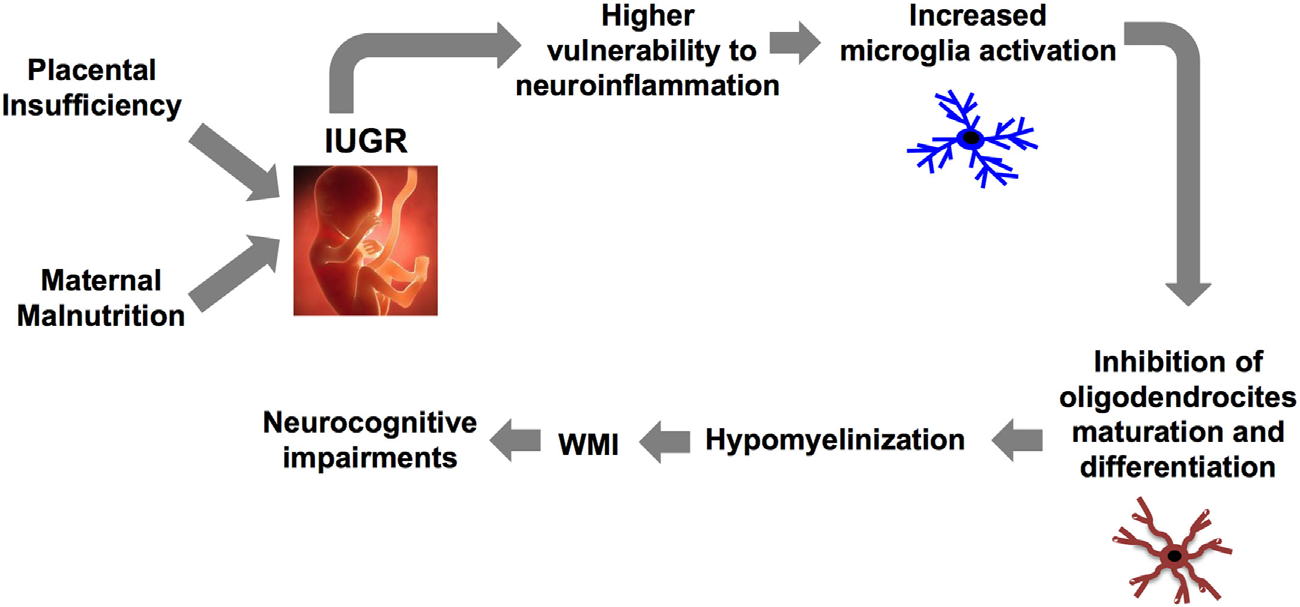
Causal relationship between abnormal microglia activation and WMI in IUGR infants. IUGR, intrauterine growth restriction; WMI, white matter injury.

The brain inflammatory response is orchestrated by crosstalk between microglia and astrocyte (26). In particular, microglia (brain resident macrophage) colonizes the brain during development in two phases: the fetal development (first two trimesters in humans and between embryonic days (EDs) 10 and 19 in rodents) and the early postnatal days (PND) (27). An accurate regulation of their activation is critical for the development of a proper immune response and for maintaining brain homeostasis. Indeed, as recently demonstrated, abnormal microglia activity can influence cortical neurogenesis (28), neuronal migration, axonal growth (29, 30), and synaptic pruning (31). These events that occur during the fetal and the early postnatal period are critical for the development of a functional brain architecture and their alterations can generate “pre-symptomatic signatures” correlated to the manifestation of neurological disease later in life as suggested by the neuro-archeological hypothesis (32). Abnormal microglia activation can also negatively affect myelinization (27). In order to better understand the relation between microglia and myelinization, it is important to consider the developmental stages of myelinization. The process is defined by initial migration and proliferation of oligodendrocyte precursors followed by their differentiation first into pre-oligodendrocytes (pre-OL) and then into mature oligodendrocytes (33). In particular, pre-OL showed higher intrinsic vulnerability to environmental insults and exposure of the brain to free radical or to excitotoxic molecules dramatically affect their maturation and differentiation (34). Abnormal microglial activation is the third factor affecting pre-OL maturation, a pivotal player in the context of WMI (27, 34) (Figure 1). Thus considering this background, the early modulation of the microglia activity could represent a valid therapeutic option for the treatment of brain injury in prematurity and to prevent the printing of the “pre-symptomatic signature” of neurological disease.

## Early Overexposure to Glucocorticosteroids (GCs): Impact on Neuroinflammation and Oxytocin Production

The release of GCs is regulated by the hypothalamic–pituitary–adrenal axis (HPA) (35). HPA axis activation results in the release of corticotropin releasing factor (CRF) from the hypothalamic paraventricular nucleus (PVN) in the portal vessel system, inducing the secretion of adrenocorticotropic hormone (ACTH) from the pituitary that in turn stimulates the release of GCs from the adrenal gland.

Glucocorticosteroids, classically described as anti-inflammatory and immunosuppressive agents, have also displayed pro-inflammatory actions. Indeed, studies conducted in humans and in rodents showed that chronic exposure to stress or to high levels of GCs potentiate the inflammatory response both at central and peripheral levels (36–38).

The pro-inflammatory effects of GCs are long lasting and early life stress is able to shift the immune response toward a pro-inflammatory phenotype later in life (39–41) with a direct effect on microglia immunoreactivity and maturation (42–45). Concerning the latter point, the study (42) showed that exposure to prenatal stress between ED 10 and 20 affects microglia maturation by inducing a reduction of immature microglia in the corpus callosum and an increase in ramified microglia in other brain regions at PND 1. More recently, two different studies demonstrated that exposure to maternal separation (MS) (43) or to prenatal stress (44) increase the activation of microglia cells in the hippocampus at PND15 and the number of activated microglia in the hippocampus and in the cortex of adult animals, respectively. In the same study (44) the authors showed, through an *in vitro* approach, that microglia isolated from prenatal stressed animals is more amoeboid and releases higher levels of pro-inflammatory cytokines. In addition, as reported in Ref. (45), exposure to prenatal stress is able to shift the hippocampal microglia morphology toward an activated phenotype not only in basal condition, but also in response to LPS stimulation in adults.

The mechanisms responsible for these effects are not yet well understood, however, an important role could be exerted by nuclear GCs receptors (GRs). GRs regulate the HPA activity by means of negative feedback (46) and the anti-inflammatory effects of GCs are promoted by the formation of a GC/GR complex (47). As previously reviewed, hippocampal GRs undergo epigenetic regulation of their expression that is influenced by early parental care (48). Interestingly, two human studies reported an increase in GR methylation in leukocytes and mononuclear cord blood cells in adults (49) and infants (50) exposed to childhood adversity, respectively. Moreover, a recent study evidenced a relation between GR methylation and inflammation at the central level (51). Rats exposed to MS showed, as adults, a higher methylation of hippocampal GR receptor that is linked to an increase in hippocampal astrocytes inflammatory response following sevoflurane administration. Interestingly, these effects can be reversed by treatment with an epigenetic regulator (51).

Because microglia are the resident immune cells of the brain, we hypothesize that stress or high levels of GCs can induce epigenetic modification of GRs on microglia too. The change in GR expression could, therefore, shift the microglia response toward the pro-inflammatory phenotype observed in premature infants and in animal models of IUGR. In this context, defining strategies to prevent exposure of the developing brain to high pro-inflammatory levels of GCs acquire greater importance.

**Figure 2:**
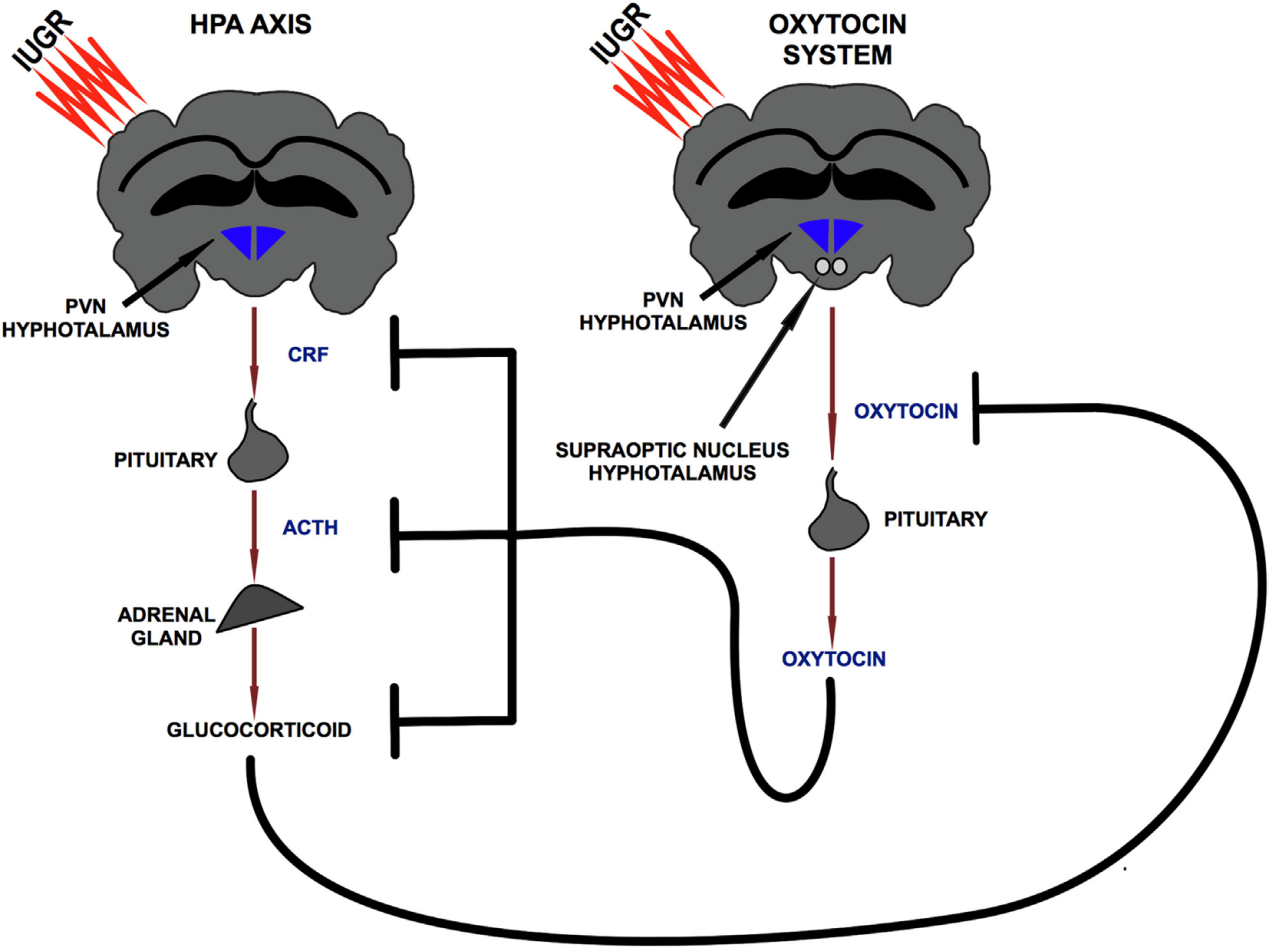
Bidirectional relationship between hypothalamic–pituitary–adrenal axis and oxytocin system. PVN, paraventricular nucleus; CRF, corticotropin releasing factor; ACTH, adrenocorticotropic hormone.

Oxytocin is a neuropeptide released by the PVN and by the supraoptic nucleus of the hypothalamus. Studies conducted in rodents and in humans showed the existence of a bidirectional relation between the HPA axis and OTX: exposure to stress induced an increase in OTX plasma levels (52–54), while OTX administration counterbalanced axis activation reducing GCs release (55–59). The details of this inhibitory action were clarified *via* pharmacological approaches in several studies. In particular, intra-cerebroventricular administration of OTX induces a reduction in CRF mRNA levels in the PVN in response to stress (55, 57) and a reduction in ACTH and corticosterone plasma levels both in the basal condition (58) and in response to stress (55, 56, 58) (Figure 2). This proven ability to modulate the GCs release supports the hypothesis of a functional interaction between the OTX system, HPA axis, and immune system. In this mutual communication between the three endogenous systems, OTX could exert an indirect anti-inflammatory action through the control of HPA axis activation. Therefore, during the early phase of life, OTX could have an important role to prevent the exposure of the brain to high and pro-inflammatory doses of GCs.

## Relation Between OTX and the Immune System: Evidences of an Anti-Inflammatory Effect

Oxytocin known for its role in labor and lactation is generally used in clinical practice for the induction and augmentation of labor (60). However, recent investigations in animals evidenced a pivotal role of OTX in the regulation of a central inflammatory response (61–63). The anti-inflammatory action in the brain was described for the first time in an animal model of brain stroke (MCAO) in combination with social housing and social isolation protocols (61). Social environment is associated with a reduction in incidence, mortality, and morbidity of stroke (61, 64). Housing in a social environment increases the synthesis of OTX mRNA in the hypothalamus and interestingly, this increase mediates the neuroprotective effects of the social environment (61). Indeed, intracerebral administration of an OTX receptor (OTXR) antagonist neutralized the neuroprotective effects of the social environment, whereas the administration of OTX to socially isolated animals before induction of cerebral arterial occlusion improved stroke outcome reducing infarct size, oxidative stress, stroke-induced gliosis, and neuroinflammation (61). In addition, a recent *in vivo* study demonstrated that intracerebral administration of OTX reduced pro-inflammatory gene expression in the hippocampus of adult animals exposed to MS (62).

Studies aimed at clarifying the cellular target of this anti-inflammatory action pointed out a role for the OTX system in the regulation of microglia reactivity both *in vivo* and *in vitro* (61, 63). Regarding the *in vivo* evidence, the study (63) demonstrated that intranasal administration of OTX to adult mice reduced microglia activation and pro-inflammatory cytokine expression induced by an LPS injection. In addition, in the same study the authors demonstrated that OXT is able to reduce the LPS-induced activation both in primary microglia and in microglia cell lines (63). Similar results have been reported in microglia cells purified from socially isolated animals and stimulated *in vitro* with LPS (61).

The biological action of OTX is linked to the activation of OTXR, a selective seven transmembrane Gq/Gi-coupled receptor (65) expressed both in astrocytes and in microglia (61, 63). Exposure of microglia cells to inflammatory stimulus induced a time-dependent increase in OTXR expression (63) suggesting that the OTX system is an inducible system that undergoes a dynamic regulation to respond to the requests of an immune challenge. The molecular bases of neuroprotective action of OTX are not well known and modulation of the downstream ERK/MAPK pathway in microglia was reported only in one study (63). In addition, other molecular effectors of OTXR (e.g., NFkB, eukaryotic elongation factor 2) could mediate the observed effect. Finally, because microglia also express receptors for glutamate and other important neurotransmitter (e.g., GABA, Acetylcholine) (66), the existence of a functional crosstalk between OTXR and other neurotransmitter receptors cannot be ruled out.

## Effects of OTX on the Neonatal Brain and “GABA Switch”

The early phase of life represents a period of maximum plasticity for the brain. Indeed, during this time it undergoes morphological changes that are fundamental for the development of correct excitatory and inhibitory neuronal circuits. An abnormal balance between excitatory and inhibitory transmission have been proposed as a causal factor for the occurrence of neurodevelopmental disorders (e.g., autism) (67) and in this context an interesting role is mediated by GABA (68, 69). GABA, the main inhibitory neurotransmitter in adults, exerts an excitatory effect in the immature brain switching transiently to an inhibitory action during delivery, and permanently during the first postnatal week (70, 71). The peculiar Cl^−^ homeostasis that characterizes the immature brain is at the base of GABA excitatory action (67). Indeed, immature neurons express on their membrane high levels of Cl^−^ importer NKCC1, and low levels of Cl^−^ exporter KCC2 with a consequent increase in Cl^−^ intracellular concentration (67). In presence of this ionic gradient, activation of the GABA receptor (GABA_A_R) induces an efflux of Cl^−^ and the consequent generation of an excitatory membrane depolarization (67).

Alteration of GABAergic signaling is reported in several neurodevelopmental diseases, such as autism (69) and Fragile X (68, 72). Therefore, the understanding of the mechanisms underlying the GABA function in the immature brain acquires greater importance.

Oxytocin is a key player for the biphasic transition of GABA and during delivery a main role is exerted by maternal OTX. Parturition is indeed associated with a massive release of OTX (73) that easily crosses the placenta and reaches the fetus (74). Combining the electrophysiological approach with *in vivo* administration of an OTXR antagonist to pregnant rats, Tyzio et al. elegantly demonstrated that maternal OTX is necessary and sufficient to promote GABA switch (70) and that the inhibition of this OTX-mediated transition induces in the offspring an autistic-like phenotype (69). The modulation of NKCC1 activity is at the base of OTX-mediated GABA switch during delivery (70) and, as observed for OTX, the administration of an NKCC1 antagonist to pregnant rats reverts the abnormal electrophysiological phenotype in two animal models of autism (69). Concerning the role of OTX in the postnatal GABA switch, a recent research highlighted the involvement of the KCC2 transporter (67). Indeed, mutant OTXR^−/−^ mice showed delayed GABA switch associated with reduced KCC2 hippocampal expression. On the contrary, wild type animals showed in the early postnatal period correct GABA transition and an increase in KCC2 expression that are promoted by activation of OTXR and of its downstream pathway Gq/protein kinase C. Interestingly, this OTXR-mediated modulation of KCC2 expression is time dependent and restricts to an early time point (67). This observation further highlighted the pivotal role of OTX in the first phase of life and supports the hypothesis of OTX as a novel neuroprotective agent in the immature brain. Indeed, a precocious treatment of preterm infants with OTX could represent a valid therapeutic strategy to ensure correct brain development and perhaps reduce the risk of developing neurodevelopmental disorders later in life.

## Effects of OTX on the Neonatal Brain and the Role of Modulation of Maternal Behavior

Oxytocin is an important hormone for the regulation of maternal behavior (75–80) and this interaction was first reported by Pedereson and Fahrbach (75, 76). Indeed, the authors demonstrated that intracerebral administration of OTX to virgin female rats reduced the latency to develop maternal care in response to exposure to forest pups (75, 76). In agreement with these results, intracerebral administration of an OTXR antagonist reduced maternal behavior and canceled the differences between high maternal care (High LG-ABN) and low maternal care (Low LG-ABN) mothers (78). High LG-ABN is associated with a higher level of OTX (77) and of OTXR in the medial preoptic area, a hypothalamic area important for the regulation of maternal care (78, 79). In humans, the increase in plasma OTX between the first and the second trimester of pregnancy is predictive of mother-infant bonding (81) and higher plasmatic and salivary levels of OTX are observed in mothers with high affectionate contact (80).

Maternal attachment is the first form of social interaction and its quality and quantity influence the behavioral and neuroendocrine outcomes of the organisms. Indeed, human studies reported that a low quantity and quality of maternal care are associated with a higher risk to develop adult psychopathy (82) and to worse cognitive performances later in life (83). In agreement with these human results, animal studies clearly demonstrated that rats reared by low LG-ABN showed, as adults, impaired cognitive performances (84, 85) increase in anxiogenic behavior (86) and fearfulness (87). Moreover, low LG-ABN showed hyperactivity of the HPA axis in response to stress (88).

Considering the ability of OTX to modulate the insurgence of maternal care and the positive effects of high levels of maternal behavior, it is possible to suggest a functional interaction between OTX and maternal care. Therefore, OTX could exert an indirect neuroprotective effect through modulation of maternal care.

## Conclusion

Intrauterine growth restriction is recognized to be an important public health problem and growth-restricted infants present an increased risk to develop cognitive and behavioral alterations later in life. As evidenced by clinical studies these increased risks are significantly correlated to the development of gray and white matter injury including altered neural circuitry. Preclinical and clinical studies have demonstrated that neuroinflammation, associated with abnormal microglia reactivity, is a causal factor for the development of WMI. Therefore, the modulation of inflammation could represent a valid therapeutic strategy for the treatment of brain injury in preterm infants. In this context, the neuropeptide OTX can exert a pivotal role due to its ability to modulate the immune system and shift its activity toward an anti-inflammatory phenotype. The studies discussed in this review demonstrate that this anti-inflammatory effect is exerted through the regulation of microglia activation. However, the beneficial effects of OTX are not only related to the modulation of neuroinflammation, but also to the development of correct neural circuitry. Indeed, its action is necessary to regulate the “GABA switch” and the proper balance between excitatory and inhibitory transmission whose alterations have been linked to the occurrence of neurodevelopmental disorders. Finally, the positive effects of OTX can not only be confined to a direct action on the immature brain. Indeed, OTX is an important regulator of maternal behavior and alterations of maternal care are correlated to the insurgence of behavioral and neuroendocrine alterations later in life. Thus, it is possible to suppose that the modulation of maternal care is one of the mechanisms at the base of OTX-mediated neuroprotection. In conclusion, the data summarized here supports the hypothesis of OTX as a potential neuroprotective agent in the developing brain.

## Methods

The present review summarizes clinical and preclinical data about causal relations between inflammation and neonatal brain injury, and recapitulates experimental evidences hypothesizing OTX as a novel anti-inflammatory and neuroprotective agent in the immature brain. A literature search was performed in December 2017–January 2018 using the PubMed library in English. No restriction of year and authors were applied and review papers were used as references only for the general concepts. The literature search relating to the pre-clinical studies was restricted to research conducted in rats and mice. Only papers that satisfied the following criteria were included: pertinence to the subject, presence of control groups, and clear descriptions of experimental procedures.

## Author Contributions

MZ and JM did the literature review. All the authors collectively analyzed articles selected in this review paper. MZ, JM, and OB wrote the manuscript. All the authors revised and approved final version of the manuscript.

## Conflict of Interest Statement

The authors declare that the research was conducted in the absence of any commercial or financial relationships that could be construed as a potential conflict of interest.
